# Fluorescent visualization and evaluation of NPC1L1-mediated vesicular endocytosis during intestinal cholesterol absorption in mice

**DOI:** 10.1093/lifemeta/load011

**Published:** 2023-03-16

**Authors:** Xiaojing Wu, Xian-Hua Ma, Jie Lin, Xiaohang Yang, Jian-Hui Shi, Zhifang Xie, Yu-Xia Chen, Weiping J Zhang

**Affiliations:** Department of Pathophysiology, Naval Medical University, Shanghai 200433, China; NHC Key Laboratory of Hormones and Development, Chu Hsien-I Memorial Hospital & Tianjin Institute of Endocrinology, Tianjin Medical University, Tianjin 300134, China; Department of Pathophysiology, Naval Medical University, Shanghai 200433, China; Department of Pathophysiology, Naval Medical University, Shanghai 200433, China; NHC Key Laboratory of Hormones and Development, Chu Hsien-I Memorial Hospital & Tianjin Institute of Endocrinology, Tianjin Medical University, Tianjin 300134, China; Department of Pathophysiology, Naval Medical University, Shanghai 200433, China; Ministry of Education Shanghai Key Laboratory of Children’s Environmental Health, Xinhua Hospital, Shanghai Jiaotong University School of Medicine, Shanghai 200092, China; Department of Pathophysiology, Naval Medical University, Shanghai 200433, China; Department of Pathophysiology, Naval Medical University, Shanghai 200433, China; NHC Key Laboratory of Hormones and Development, Chu Hsien-I Memorial Hospital & Tianjin Institute of Endocrinology, Tianjin Medical University, Tianjin 300134, China

**Keywords:** cholesterol homeostasis, NPC1L1-EGFP, cholesterol transportation, vesicle endocytosis, CRISPR/Cas9, knock-in mice

## Abstract

Excessive cholesterol absorption from intestinal lumen contributes to the pathogenesis of hypercholesterolemia, which is an independent risk factor for atherosclerotic cardiovascular disease. Niemann-Pick C1-like 1 (NPC1L1) is a major membrane protein responsible for cholesterol absorption, in which the physiological role of vesicular endocytosis is still controversial, and it lacks a feasible tool to visualize and evaluate the endocytosis of NPC1L1 vesicles *in vivo*. Here, we genetically labeled endogenous NPC1L1 protein with EGFP in a knock-in mouse model, and demonstrated fluorescent visualization and evaluation of the endocytic vesicles of NPC1L1-cago during intestinal cholesterol absorption. The homozygous NPC1L1-EGFP mice have normal NPC1L1 expression pattern as well as cholesterol homeostasis on chow or high-cholesterol diets. The fluorescence of NPC1L1-EGFP fusion protein localizes at the brush border membrane of small intestine, and EGFP-positive vesicles is visualized beneath the membrane as early as 5 min post oral gavage of cholesterol. Of note, the vesicles colocalize with the early endosomal marker early endosome antigen 1 (EEA1) and the filipin-stained free cholesterol. Pretreatment with NPC1L1 inhibitor ezetimibe inhibits the formation of these cholesterol-induced endocytic vesicles. Our data support the notion that NPC1L1-mediated cholesterol absorption is a vesicular endocytic process. NPC1L1-EGFP mice are a useful model for visualizing cellular NPC1L1-cargo vesicle itineraries and for evaluating NPC1L1 activity *in vivo* in response to diverse pharmacological agents and nutrients.

## Introduction

Cholesterol is an essential structural component of mammalian cell membrane and plays important roles in cell signaling transduction, intracellular transport, and the synthesis of steroid hormones and bile acids [1]. Clinical studies have revealed that high levels of plasma cholesterol are an independent risk factor for atherosclerotic cardiovascular disease, the leading cause of death in developed countries [2–4].

Cholesterol homeostasis is mainly maintained by intestinal absorption, *de novo* synthesis, and biliary and transintestinal secretion [1]. The absorption of cholesterol in small intestine is primarily mediated by Niemann-Pick C1-like 1 (NPC1L1) protein [5, 6], an extensively N-glycosylated protein composed of 1332 amino acids with 13 transmembrane segments [7]. It has a typical N-terminal signal peptide, which targets the protein to cell membranes. The N-terminal extracellular segment of the protein can selectively bind cholesterol and sitosterol, and a less conserved C-terminal cytoplasmic tail bears an endocytic signal sequence essential for internalization of the protein-cargo complex [7–10]. The expression pattern of this protein is species and tissue specific. In rodents such as mice, NPC1L1 protein is almost exclusively expressed in the brush border membrane of small intestine, responsible for cholesterol intake from gut lumen [5, 6, 11]. In humans and non-human primates, NPC1L1 also localizes on the canalicular membrane of the hepatocytes, responsible for re-absorbing cholesterol from bile back to hepatocytes [12]. The mRNA expression of NPC1L1 is influenced by cholesterol and a variety of unsaturated fatty acids, which involves several nuclear receptors. For example, NPC1L1 mRNA levels are reduced by addition of cholesterol through sterol regulatory element binding protein 2 (SREBP2) and increased by depletion of cholesterol in human Caco-2 colon cancer cell and Huh7 hepatoma cell lines [13, 14]. SREBP2 directly binds to the sterol response element (SRE) located at −748/−738 bp and −91/−81 bp in the human NPC1L1 promoter [14]. In cultured cells, the subcellular location of NPC1L1 protein is also regulated by cholesterol with the protein predominantly localized at the transferrin-positive and cholesterol-rich endocytic recycling compartment (ERC) when cellular cholesterol is abundant; the protein is translocated to the plasma membrane when cellular cholesterol is low, and this translocation to the plasma membrane is coupled to NPC1L1-dependent cholesterol uptake [15].

NPC1L1 knockout mice are resistant to diet-induced hypercholesterolemia [5], and their cholesterol absorption rate decreases by about 70%, while the absorption of other lipids (such as triglycerides and phospholipids) is not affected, providing the compelling evidence that NPC1L1 specifically mediates the absorption of cholesterol [5, 6]. It appears that NPC1L1 also mediates intestinal absorption of non-cholesterol sterols such as phytosterols, as ezetimibe treatment can effectively reduce phytosterol accumulation in humans and animals with sitosterolemia [16]. When there is a high concentration of cholesterol in the lumen, the plasma membrane-localized NPC1L1 binds cholesterol via its N-terminal domain, and its C-terminal tail dissociates from plasma membrane, exposing the endocytic signal peptide (YVNxxF) available for the clathrin adaptor NUMB protein recognition [1, 10]. The scaffold protein clathrin and its adapter protein 2 (AP2) are then recruited to assemble endocytic vesicles and transported to ERC along the microfilament [10, 17]. After completing cholesterol unloading, NPC1L1 is recognized by LIM domain and actin binding 1 (LIMA1) protein through the QKR sequence at the C-terminus, and then recycled to plasma membrane for reuse with the help of small G protein cell division cycle 42 (Cdc42) and its downstream proteins neural Wiskott-Aldrich syndrome protein (N-WASP), Arp2/3, and myosin Vb [18–20]. It was demonstrated that ezetimibe, a small molecule compound used in the treatment of hypercholesterolemia, inhibits cholesterol absorption by binding to the second extracellular loop region of NPC1L1 to block the formation of endocytic vesicles [11, 17, 21, 22].

Although the role of NPC1L1 in intestinal cholesterol absorption has been well established, most mechanistic studies were performed in rat or human hepatoma cells *in vitro* [10, 17, 20, 23], and it remains controversial whether NPC1L1-mediated cholesterol absorption is a vesicular endocytic process [23]. In the present study, we genetically engineered the endogenous *Npc1l1* gene to express a fusion protein with enhanced green fluorescent protein (EGFP) in mice, enabling us to dynamically visualize the intracellular trafficking of NPC1L1 protein during intestinal absorption of dietary cholesterol. Our data demonstrated that cholesterol-induced endocytosis of NPC1L1-positive vesicles is involved in intestinal cholesterol absorption and this endocytic process can be blocked by ezetimibe.

## Results

### Generation of NPC1L1-EGFP knock-in mouse model

**Figure 1:**
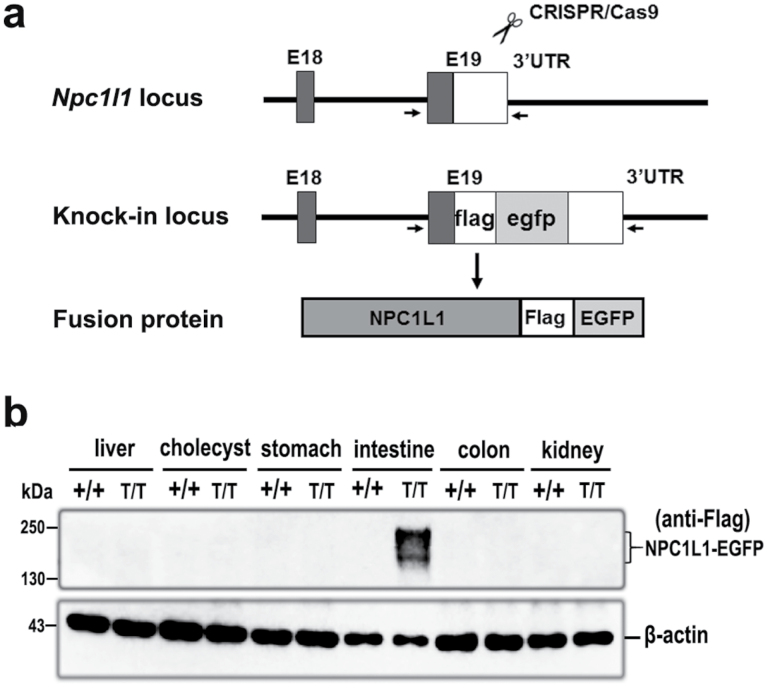
Generation of NPC1L1-EGFP mice. (a) Schematic diagram for knock-in of FLAG/EGFP dual tags in the C-terminal end of NPC1L1. (b) Tissue distribution of NPC1L1-EGFP protein. Eight-week-old male mice were sacrificed and tissues were collected for SDS-PAGE followed by immunoblotting with anti-FLAG-M2 or anti-β-actin antibody. Immunoblots are representative of three independent experiments.

To genetically label endogenous NPC1L1 protein with FLAG tag and EGFP, we took advantage of CRISPR/Cas9 technology to introduce the FLAG-EGFP-encoding sequence immediately upstream the stop codon of mouse *Npc1l1* gene to express NPC1L1-FLAG-EGFP fusion protein (hereafter, NPC1L1-EGFP) (Fig. 1a). Western blot analysis using anti-FLAG M2 antibody showed that NPC1L1-EGFP protein was detected in the small intestine from the homozygous NPC1L1-EGFP mice (*Npc1l1*^T/T^, hereinafter T/T), but not in the liver, kidney, stomach, or gallbladder (Fig. 1b). The expression levels of NPC1L1 in mRNA and protein were not significantly different in the jejunum between T/T and control mice (Supplementary Fig. S1).

### Characterization of NPC1L1-EGFP protein expression and distribution

**Figure 2:**
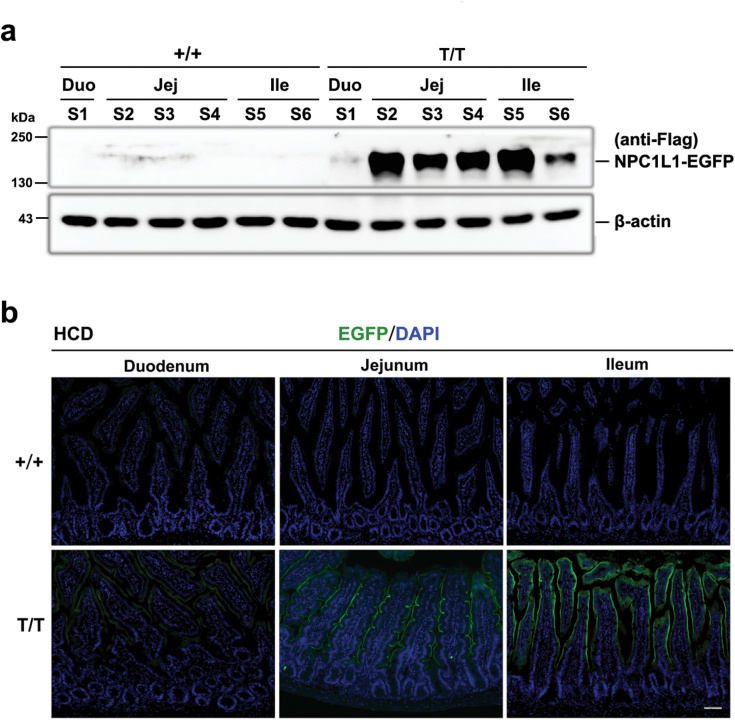
Distribution of NPC1L1-EGFP protein in the small intestine. The small intestine of 8-week-old male mice was removed and divided into 6 segments of equal length. (a) All tissues were immediately homogenized and subjected to SDS-PAGE followed by immunoblotting with anti-FLAG-M2 or anti-β-actin antibody. Immunoblots are representative of at least three independent experiments. (b) Localization of NPC1L1-EGFP protein along the duodenum-ileum axis. S1 (duodenum), S3 (jejunum), and S5 (ileum) segments were fixed with 2% PFA and stained with DAPI for visualization of EGFP-fused NPC1L1 protein. Scale bar: 50 µm.

We further characterized the expression pattern of NPC1L1-EGFP fusion protein in intestine by western blot analysis. Abundant expression of the fusion protein was detected in the jejunum and proximal half of ileum (intestinal segments S2–S5), and to much lesser extent, in duodenum and distal half of ileum (Fig. 2a). We then observed the distribution of NPC1L1 protein in the villous epithelia of small intestine. Fluorescence microscope examination of frozen sections revealed robust EGFP fluorescence outlining clearly the brush border membrane of the villus rather than the crypts in the duodenum, jejunum, and ileum of T/T mice (Fig. 2b). On the contrast, NPC1L1 expression was undetectable in the colon (Supplementary Fig. S2). This expression pattern is consistent with the characteristics of endogenous NPC1L1 distribution in mice as previously reported [5, 11, 18]. These data suggest that EGFP or FLAG can be used as a surrogate marker for endogenous NPC1L1 protein in NPC1L1-EGFP mice.

### Characterization of cholesterol metabolism in NPC1L1-EGFP mice

**Figure 3:**
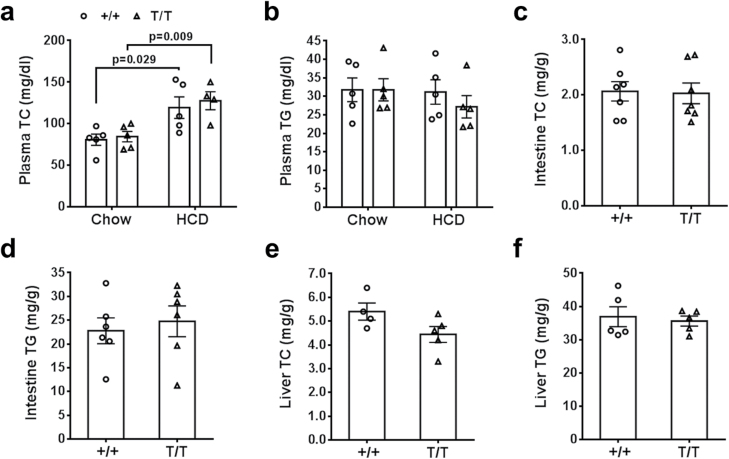
Lipid metabolism in NPC1L1-EGFP mice. Two-month-old NPC1L1-EGFP knock-in mice (T/T) and the control mice (+/+) were fed high-cholesterol diet (HCD) for 3 weeks. TC and TG in the plasma (a and b), intestine (c and d), and liver (e and f) were detected.

To address whether the fusion with FLAG-EGFP could affect NPC1L1 protein function, we analyzed cholesterol metabolism of the knock-in mice. On normal chow diet, there was no difference in plasma levels of total cholesterol (TC) or total triglycerides (TG) between control and homogenous T/T mice under fed condition (Fig. 3a and b). When switched to high-cholesterol diet (HCD, 1.25% cholesterol) for 3 weeks, the knock-in and control mice exhibited similar body weight, food intake (Supplementary Fig. S3a and b), and plasma TC and TG levels, although as expected, their plasma TC levels increased significantly compared to the chow-fed mice (Fig. 3a and b). Furthermore, HCD-fed T/T mice did not show any significant gross or morphological changes in the small intestine and liver compared to the control (Supplementary Fig. S3c–e). Of note, there was no significant difference in intestinal and hepatic contents of TC and TG between the two genotypes of HCD-fed mice (Fig. 3c–f). These results suggest that the C-terminal fusion with FLAG-EGFP does not affect the activity of NPC1L1 protein in cholesterol metabolism, which is consistent with previous reports with hepatoma cells *in vitro* [15, 25].

### Visualization of cholesterol-induced NPC1L1 vesicle trafficking

**Figure 4:**
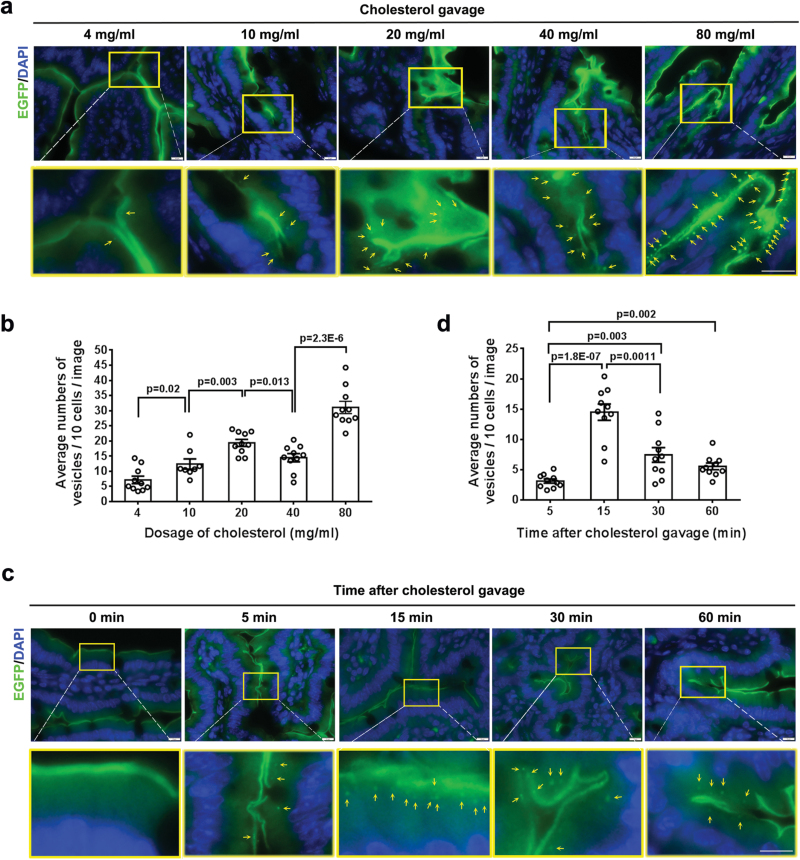
Visualization of endocytic vesicles in the intestine induced by cholesterol in T/T mice. (a and c) The T/T mice were fasted overnight and then were administrated by gavage with 200 µL corn oil containing different concentrations of cholesterol for 15 min (a), or 200 µL corn oil containing 40 mg/mL cholesterol for 0–60 min (c). The mice were then sacrificed at the indicated time for the small intestine fluorescence studies (S3–S4). (b and d) Average numbers of NPC1L1-EGFP containing vesicles beneath the brush border membrane of the jejunum. Multiple images (*n* = 8–10) were taken and counted from per mouse (*n* = 2–3). Scale bar: 10 µm.

**Figure 5:**
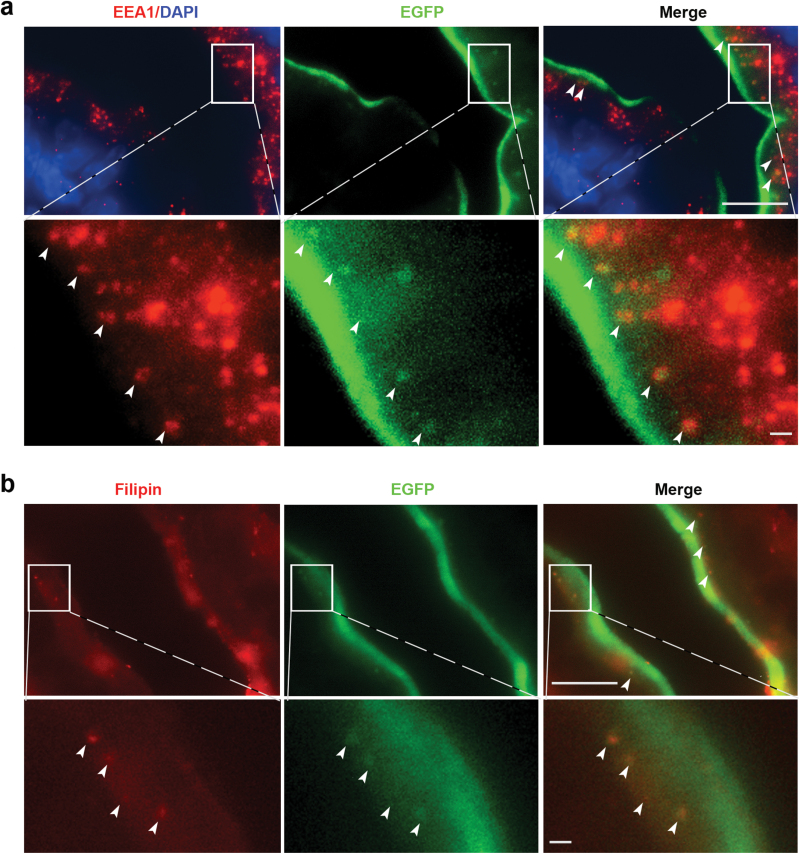
Co-localization of NPC1L1-EGFP vesicles with EEA1 and cholesterol. The T/T mice were fasted overnight and then were administrated by gavage with 200 µL corn oil with or without 80 mg/mL cholesterol for 15 min. Five micrometer cryo-sections of jejunum (S3) were prepared for co-localization assay. (a) Immuofluorescence detection of NPC1L1-EGFP vesicles colocalized with the endosomal marker EEA1. (b) The sections were stained with 50 µg/mL filipin to visualize the free cholesterol in the NPC1L1-EGFP vesicles. Scale bar: 10 µm; inner scale bar: 1 µm.

We next sought to visualize NPC1L1 internalization in intestinal cholesterol absorption in NPC1L1-EGFP mice. To capture the appropriate time window for tissue sampling, we first dynamically monitored the movement of luminal contents in small intestine by oral gavage of 200 μL of Chinese ink. In overnight-fasted mice, the leading edge of black ink reached S4 (jejunum) at 5 min, S5 (proximal half of ileum) at 15 min, and S6 (distal half of ileum) at 30 min after the gavage (Supplementary Fig. S4), respectively. We then administered 200 μL of corn oil containing 4–80 mg/mL cholesterol by oral gavage in overnight-fasted T/T mice, and sampled the jejunum S4 at different time points to prepare cryo-sections for fluorescence microscopic examination. Fifteen minutes after cholesterol gavage, there was dose-dependent increase in the number of EGFP-positive vesicles beneath the brush border membrane in the jejunum (Fig. 4a), with the most abundant signals elicited by 80 mg/mL of cholesterol (Fig. 4b). Of note, EGFP-positive vesicles could be observed beneath the brush border membrane of jejunum as early as 5 min after gavage of 40 mg/mL cholesterol (Fig. 4c), with the vesicles number peaking at 15 min and greatly declining at 60 min (Fig. 4d). Immunohistochemical staining revealed that some EGFP-positive vesicles were colocalized with early endosome antigen 1 (EEA1), an early endosomal marker (Fig. 5a and Supplementary Fig. S5a), indicating a nature of NPC1L1 endocytic vesicles. Furthermore, filipin staining showed some EGFP-positive vesicles were loaded with cholesterol (Fig. 5b and Supplementary Fig. S5b). These data suggest that our mouse model enables us to fluorescently visualize and evaluate intestinal cholesterol absorption via the endocytosis of NPC1L1-cargo.

### Blockade of cholesterol-induced NPC1L1 endocytosis by ezetimibe

**Figure 6:**
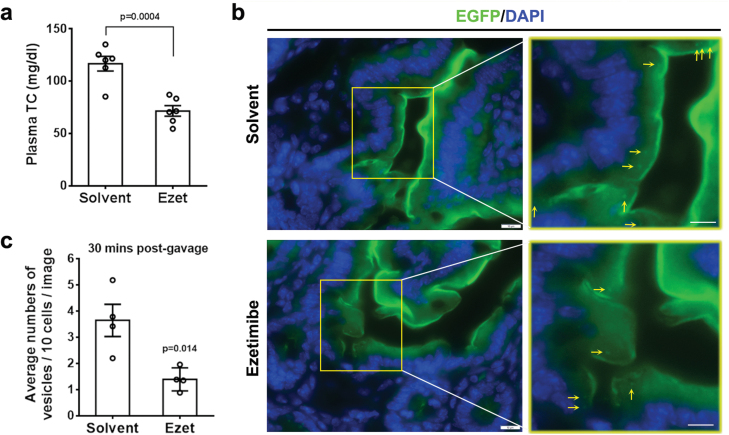
Ezetimibe inhibits the transport of cholesterol-containing endocytic vesicle. (a) Four-month-old male mice were pretreated with solvent or ezetimibe (10 mg/kg/day) by gavage for 5 days. On day 6, the overnight-fasted mice were treated with ezetimibe or solvent. After 50 min, the mice were administered by gavage with 200 μL corn oil containing 40 mg/mL cholesterol. The mice were sacrificed 30 min post gavage for fluorescence studies (S4). (b) NPC1L1-EGFP mice were treated by daily gavage administration of ezetimibe (10 mg/kg/day) for consecutive 14 days, and plasma were then analyzed for TC concentration. (c) Average numbers of NPC1L1-EGFP containing vesicles. Multiple images (*n* = 10) were taken and counted from per mouse (*n* = 4). Scale bar: 10 µm.

We further sought to verify whether ezetimibe inhibits cholesterol-induced vesicle endocytosis. To this aim, we first tested the cholesterol-lowering effect of ezetimibe in T/T mice. Two weeks of ezetimibe administration by gastric gavage at 10 mg/kg led to an ~39% decrease in plasma TC levels, suggesting the dose of ezetimibe could inhibit intestinal cholesterol absorption (Fig. 6a). Then we subjected T/T mice to gastric gavage of the same dose of ezetimibe for 5 days prior to fluorescent visualization of NPC1L1 vesicles. Ezetimibe treatment dramatically reduced the number of EGFP-positive NPC1L1 endocytic vesicles beneath the brush border membrane in jejunum S4 after 30 min of cholesterol gavage (Fig. 6b and c). These data support the notion that ezetimibe inhibits NPC1L1 endocytosis and this inhibition may, at least in part, contribute to the cholesterol-lowering effect of ezetimibe.

## Discussion

NPC1L1 plays an important role in the absorption of cholesterol, other closely related sterols such as sitosterol and tocopherol. The present study establishes a mouse model to visualize and evaluate the vesicle endocytosis of NPC1L1-cargo during intestinal cholesterol absorption, eliminating the need of immunostaining. Although the C-terminus of NPC1L1 triggers cholesterol endocytosis [10, 18], genetically engineering of the endogenous NPC1L1 via C-terminal fusion with EGFP does not significantly affect cholesterol homeostasis under normal chow diet or HCD conditions. The expression of NPC1L1-EGFP fusion protein is only detected in the small intestine, with the highest abundance in jejunum and ileum, which is consistent with the expression pattern of endogenous NPC1L1 protein [5, 11, 18]. Under fluorescence microscope, the NPC1L1-EGFP fusion protein localizes on the brush border membrane of enterocytes, and internalized in the form of endocytic vesicles beneath the membrane as early as 5 min after oral gavage of cholesterol. Moreover, the cholesterol-induced NPC1L1-EGFP endocytosis in enterocytes is markedly inhibited by ezetimibe, an inhibitor of cholesterol absorption. Thus, the NPC1L1-EGFP knock-in mice provide a useful tool to visualize and evaluate NPC1L1 endocytosis during intestinal cholesterol absorption.

It is putatively regarded that NPC1L1 intakes extracellular cholesterol through formation of endocytosis vesicle assembled by clathrin/AP2 complex, which is largely based on *in vitro* experiments using rat and human hepatoma cells overexpressing NPC1L1-EGFP [17]. This is also supported by the observation in the transgenic mice harboring human NPC1L1, which reveals the formation of NPC1L1-vesicles beneath the brush border membrane of the jejunum after cholesterol intake by immunofluorescence histochemistry [11, 26]. In addition, ezetimibe shows inhibitory effect on the internalization of NPC1L1 as well as intestinal cholesterol absorption in the mice, the latter of which is evidenced by filipin staining [17]. However, this notion was challenged by a study, which raises the possibility that NPC1L1-mediated cholesterol uptake could be independent of NPC1L1 endocytosis [23]. By cell surface biotinylation assay of the same hepatoma cell model, Johnson *et al.* reported that ezetimibe could inhibit cholesterol binding to NPC1L1 but not NPC1L1 internalization, and the endocytosis inhibitors (Dyngo-4a or Pitstop 2) blocked completely NPC1L1 endocytosis but not ezetimibe-sensitive cholesterol uptake. In our NPC1L1-EGFP mice, we observed the formation of NPC1L1-positive vesicles as early as 5 min after cholesterol gavage, a process could be inhibited upon ezetimibe pretreatment. Notably, using the intestinal cryo-sections, we presented firstly the *in vivo* co-localizations of the endosomal marker EEA1 and cholesterol with NPC1L1-positive vesicles. Thus, our data suggest that NPC1L1-mediated cholesterol absorption in the intestine is most likely dependent on the endocytosis under the physiological condition.

In summary, the NPC1L1-EGFP mice enable us to visualize the endocytosis and trafficking of endogenous NPC1L1 vesicles, thereby providing a useful tool to evaluate intestinal cholesterol and tocopherol absorption under pathophysiological and pharmacological conditions.

## Materials and methods

### Animals

*Npc1l1-EGFP* knock-in mice were generated by CRIPRS/Cas9 in Biocytogen (Beijing, China) with the genetic background of C57BL/6N. All mice were housed in a specific pathogen-free animal facility at 25°C with a daylight cycle from 8 a.m. to 8 p.m. The homozygous male mice aged 8–12 weeks were used in the experiments.

### Mouse genotyping

Mouse genotyping was performed by PCR analysis of tail genomic DNA, with the primers of 5ʹ-CAGGGCCAGATGTTAACCAAGCTCT-3ʹ, and 5ʹ-GTACCACTGCCACACGTTCCCAAG-3ʹ. The reaction conditions were 32 cycles of 95°C (15 s), followed by 62°C (20 s) and 72°C (1 min), and a final extension for 7 min at 72°C. The PCR products were used for 2% agarose gel electrophoresis to analyze the genotype according to the size of the products.

### Quantitative real-time PCR

The total RNA was extracted from intestine by Trizol according to the product introduction. One microgram RNA was used for cDNA synthesis and the relative mRNA level of indicated genes were analyzed by the ΔΔCT method. *Actb* was used as internal control. The primers used were sense 5ʹ-CTGCCCCCACCGAAACAAAAAGAA-3ʹ, antisense 5ʹ-GCGGCAGCAGGAGGAGGATGG-3ʹ for mouse *Npc1l1*, and sense 5ʹ-CCCTAAGGCCAACCGTGAAAAGAT-3ʹ, antisense 5ʹ-ACCGCTCGTTGCCAATAGTGATGA-3ʹ for mouse *Actb*.

### Western blot

After anesthesia with 4% chloral hydrate (0.1 mL/kg), the stomach, small intestine, liver, gallbladder, kidney, and other tissues were frozen in liquid nitrogen. Whole tissue lysates were extracted from 30 mg of various tissues in 0.5 mL urea lysis buffer (8 mol/L urea, 0.1 mol/L Tris-HCl, pH 8.0), then separated on 8% SDS-PAGE gel electrophoresis, and transferred to PVDF membrane. The blots were incubated with anti-FLAG M2 antibody (1:1000, Abmart, M20008), anti-NPC1L1 antibody (1:1000, Novus, NB400-128), and anti-β-actin antibody (1:2000, ProteinTech, #66009-1), respectively, overnight at 4°C before incubation with HPR-conjugated secondary antibody and subsequent development.

### Visualization of NPC1L1 protein and endocytic vesicles

After the mice were anesthetized by inhalation of 3.5% isoflurane, the small intestine tissues were quickly removed and separated into 6 equal segments (S1: duodenum, S2–S4: jejunum, S5–S6: ileum). 0.5 cm of each middle segment was cut, immersed in 2% neutral paraformaldehyde, and fixed 1 h at room temperature. After washing with PBS, the tissues were dehydrated overnight in 15% and 30% sucrose solution sequentially, then embedded in Tissue-Tek OCT (Sakura, 4583), and frozen in liquid nitrogen. The sections with a thickness of 5 µm were dried and stained with 10 µg/mL DAPI in PBS for 5 min before mounting. The fluorescence images were captured using a fluorescence microscope equipped with a digital camera (Olympus BX53/DP80).

For quantification of intracellular EGFP-positive vesicles, 10 images of fluorescence pictures with 2.2 µm thickness were randomly taken at 100× magnification on confocal microscope (Olympus Fluoview FV3000). EGFP-positive vesicles and DAPI-labeled nucleus were counted using CellSens Dimension software. The final numbers of vesicles were normalized with cell numbers per frame.

### Intestinal filipin staining

The intestinal cryo-sections of 5 μm thickness were stained with 50 μg/mL filipin diluted in 1 × PBS for 30 min at 37°C and mounted in cover slips after 2 times of wash in 1 × PBS. Intracellular free cholesterol stained with filipin was imaged by Lionheart FX automated microscopy (BioTek).

### Immunofluorescence

The frozen sections of the intestine were prepared as described above. For EEA1 staining, 5 μm cryo-sections were incubated with rabbit anti-EEA1 polyclonal antibody (Proteintech) overnight at 4°C. After 3 times of wash with PBS, Alexa Fluor 594-conjugated goat anti-rabbit secondary antibody (Invitrogen) was sequentially added and incubated for 1 h at 37°C. After immunostaining, sections were washed in PBS for 3 times before mounting. Fluorescence pictures were taken under Lionheart FX automated microscopy (BioTek).

### Lipid analysis in plasma, liver, and intestine

After fed 1.25% HCD (D12108C, Research Diets, New Brunswick, NJ) for 3 weeks, mice were fasted for 6 h and euthanized. The plasma was collected and the lipids were extracted from liver and jejunum with acetone for colorimetric assays of the concentrations of TC and TG as described previously [24].

### Statistical analysis

All values unless otherwise indicated are expressed as mean ± SEM. Statistical analyses were carried out using Student’s *t* test or ANOVA followed by *post hoc* comparisons, and differences were considered significant when *P *< 0.05.

## Supplementary Material

load011_suppl_Supplementary_Material

## Data Availability

Most data are included in the article and in the online supplementary data. Additional data underlying this article may be obtained on reasonable request to the corresponding author.
