# Supplementary material for: Fluorescent visualization and evaluation of NPC1L1-mediated vesicular endocytosis during intestinal cholesterol absorption in mice
Source: Life Metab. 2023 Mar 16;2(2):load011. doi: 10.1093/lifemeta/load011 (PMC11749127; doi:10.1093/lifemeta/load011)
Supplement: load011_suppl_Supplementary_Material [file load011_suppl_Supplementary_Material.docx]

**

**

**Supplementary Figure 1. NPC1L1 mRNA and protein expressions in the small intestine of NPC1L1-EGFP mice.** The small intestine was harvested from male control (+/+) and NPC1L1-EGFP transgenic mice (T/T) at the age of 8 weeks. a: The level of NPC1L1 mRNA in the S3 segments was determined by quantitative RT-PCR (n=6). b: The expression of NPC1L1 protein was detected by western blotting with anti-NPC1L1 antibody (Novus). S1: duodenum, S2-4: jejunum, S5-6: ileum. NPKO, NPC1L1 knockout. Arrow indicates the non-specific bands in the +/+ mice.


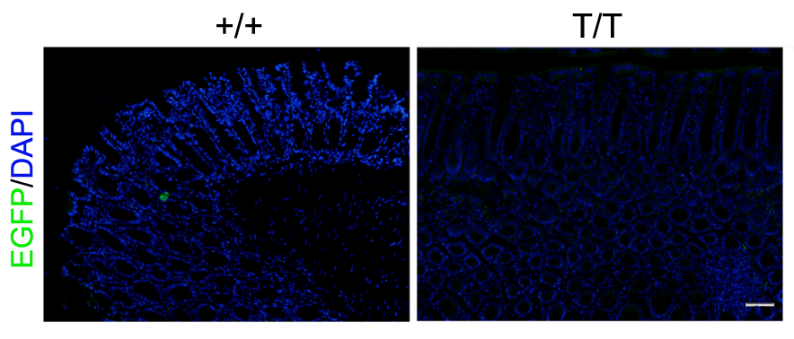


**Supplementary Figure 2. Absence of NPC1L1-EGFP protein expression in the colon from NPC1L1-EGFP mice.** The colon was harvested from male control and NPC1L1-EGFP transgenic mice (T/T) at the age of 8 weeks, fixed with 2% PFA, and stained with DAPI for visualization of EGFP-fused NPC1L1 protein. Scale bar: 50 µm.


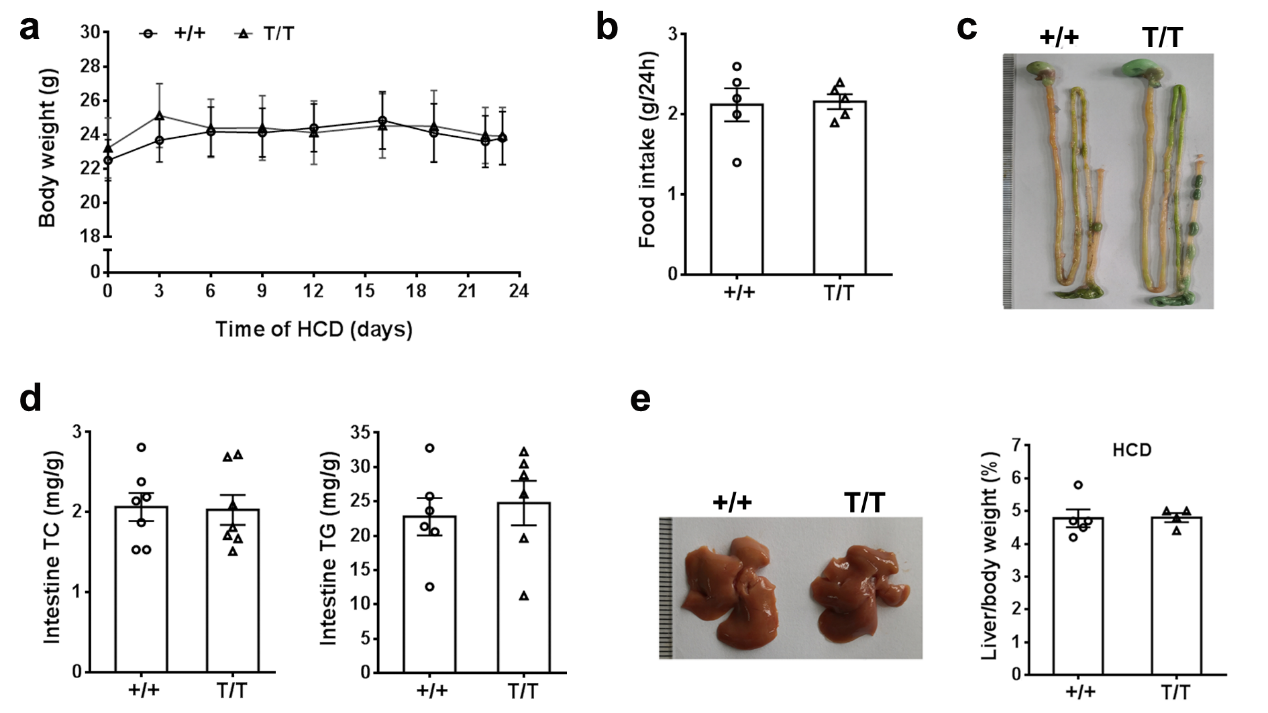


**Supplementary Figure 3. lipid metabolism in the NPC1L1-EGFP mice.** Two-month-old mice were fed high cholesterol diet (HCD) for 3 weeks. The NPC1L1-EGFP knock-in mice (T/T) showed the similar phenotype with the wild type mice (+/+) in body weight (a), food intake (b), intestine appearance (c), intestine TC and TG (d), liver appearance and weight (e).


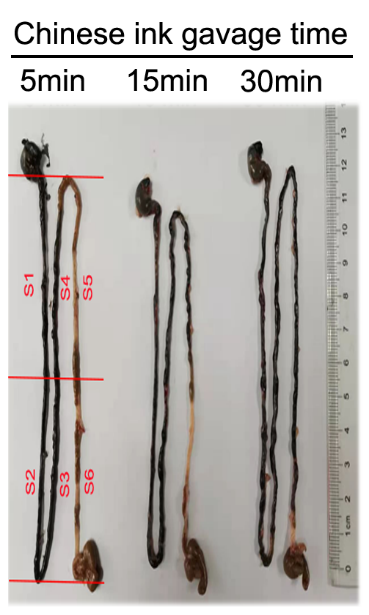


**Supplementary Figure 4. The small intestine propulsion assay in the NPC1L1-EGFP mice.** The T/T mice were fasted overnight and then were administrated by gavage with 200 µl Chinese ink for 5-30 min. The mice were then sacrificed at the indicated time for the observation of the front edge of ink propulsion in the small intestine.


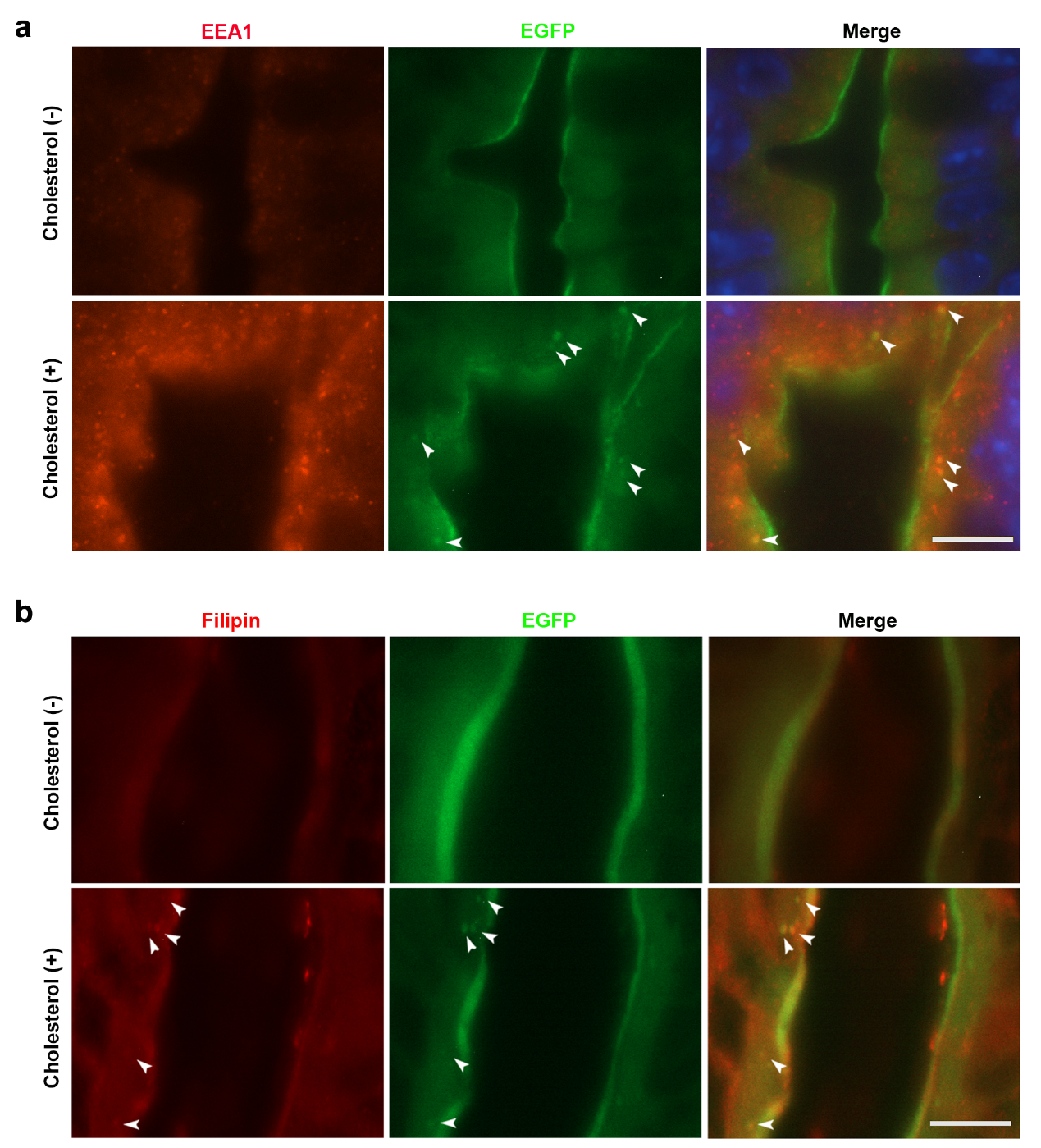


**Supplementary Figure 5. NPC1L1-EGFP vesicles co-localize with EEA1 and filipin.** The T/T mice were fasted overnight and then were administrated by gavage with 200 µl corn oil with or without 80 mg/ml cholesterol for 15 min. 5µm cryo-sections of jejunum (S3) were prepared to immunostain with anti-EEA1 antibody (a) or stain with 50 µg/ml filipin (b). Arrows in the Merge images indicate the stronger localization of EEA1 (a) and filipin (b) in the NPC1L1-EGFP vesicles. scale bar: 10 µm.
